# Ecological Predictors of Older Adults’ Participation and Retention in a Physical Activity Intervention

**DOI:** 10.3390/ijerph19063190

**Published:** 2022-03-08

**Authors:** Manuela Peters, Tiara Ratz, Frauke Wichmann, Sonia Lippke, Claudia Voelcker-Rehage, Claudia R. Pischke

**Affiliations:** 1 Leibniz Institute for Prevention Research and Epidemiology—BIPS, 28359 Bremen, Germany; wichmann@leibniz-bips.de; 2 Department of Psychology & Methods, Jacobs University Bremen, 28759 Bremen, Germany; t.ratz@jacobs-university.de (T.R.); s.lippke@jacobs-university.de (S.L.); 3 Department of Neuromotor Behavior and Exercise, Institute of Sport and Exercise Sciences, University of Muenster, 48149 Muenster, Germany; claudia.voelcker-rehage@uni-muenster.de; 4 Institute of Medical Sociology, Centre for Health and Society, Medical Faculty, Heinrich Heine University Duesseldorf, 40225 Duesseldorf, Germany; claudiaruth.pischke@med.uni-duesseldorf.de

**Keywords:** response, dropout, older adults, physical activity interventions, OSM, GIS

## Abstract

Research is still lacking regarding the question as to how programs to promote healthy ageing should be organized in order to increase acceptance and thus effectiveness. For older adults, ecological factors, such as the physical distance to program sites, might predict participation and retention. Thus, the key aim of this analysis was to examine these factors in a physical activity intervention trial. Adults (*N* = 8299) aged 65 to 75 years were invited to participate and *n* = 589 participants were randomly assigned to one of two intervention groups with 10 weeks of physical activity home practice and exercise classes or a wait-list control group. Response, participation, and dropout data were compared regarding ecological, individual, and study-related variables. Kaplan–Meier curves and Cox regression models were used to determine predictors of dropout. In total, 405 participants completed the study. Weekly class attendance rates were examined regarding significant weather conditions and holiday periods. The highest rates of nonresponse were observed in districts with very high neighborhood levels of socioeconomic status. In this study, ecological factors did not appear to be significant predictors of dropout, whereas certain individual and study-related variables were predictive. Future studies should consider these factors during program planning to mobilize and keep subjects in the program.

## 1. Introduction

Despite the strong evidence of physical and psychological health benefits of physical activity (PA) [1,2,3], PA remains a leading cause of morbidity and mortality worldwide [4]. As overall life expectancy has increased during the past decades, population-based approaches that target older people in fostering the maintenance of health-promoting behaviors, including regular moderate to vigorous PA, are called for [5]. Less than half of German older adults meet required PA levels [6] based on the recommendations provided by the World Health Organization (WHO) for adults aged 65 years and above [7]. Unfortunately, the majority of interventions fail to ensure that a physically active lifestyle and attendance of PA programs is maintained for extended periods of time [8]. Another challenge is to get the target group involved in intervention programs in the first place. Many researchers examining community-based programs for health promotion face difficulties in recruiting and retaining participants in their trials [9]. Certain barriers to recruitment may arise with respect to studies designed to involve older adults, such as issues around the identification of appropriate individuals and physical access. Non-respondents may systematically differ from respondents in certain baseline characteristics [10] (e.g., more health-conscious individuals may be more likely to self-select to PA programs and trials [11]). Keeping participants in research programs and thus obtaining an adequate amount of outcome data is another major challenge [10]. Attrition rates, in particular, in e-health interventions are high, ranging from 50–80% [10,12]. Another point of concern is the possibility of selective dropout (e.g., higher attrition in either the intervention group (IG) or the control group (CG) [13]). Further, adherence to interventions is important for the intervention’s impact on health outcomes. However, in addition to dropout and partial loss of participants, suboptimal recruitment that misses out the full range of the target population can be a problem as conclusions about the effectiveness of programs might be skewed. Also, corresponding research trials can cause biased results [14] and consequently impact the external validity of the study and the generalizability of the findings to the general population [10,15]. Thus, to increase the effectiveness of health interventions and to attract older adults to PA interventions and ensure completion of these programs, a better understanding of factors that act as barriers or facilitators for participation and retention are fundamental. 

There are several factors known to affect participation and retention in PA intervention programs among older adults, including individual-level factors, ecological factors and those related to the study and program design [16,17]. A framework related to the social dimension is the socioemotional selectivity theory (STT) by Carstensen et al. which describes the relative importance and changes of personal life goals for different age groups, stating that older adults have more goals associated with emotions than younger age groups [18]. In its core, the theory posits that when lifetime limitations are perceived, present-oriented goals with emotional significance (e.g., intensifying social contacts) are prioritized over future-oriented goals, such as expanding individual knowledge. There are indications that psychosocial factors (including knowledge, beliefs, self-efficacy and social support) play a role in older adults’ behavior, such as participation in interventions and adherence to community exercise classes [16]. Based on a systematic review, there is evidence suggesting health interventions may be particularly successful, if they are grounded in social cognitive models that address psychological factors such as the perceived barriers of preventative health behavior [19]. However, such models have been criticized for being better at predicting behavioral intention than actual behavior [19]. Therefore, it is important to consider a wider perspective regarding potential influencing factors. Thus, a broader theoretical basis for the multidimensional analysis presented in this article is based on socio-ecological approaches to promote active living, which consider the complex interrelations between individuals, social and physical environments, including organizational factors [20,21,22].

In the following, empirical evidence on the supposed individual and ecological factors influencing participation and retention is presented based on a literature review.

## 2. Literature Review

### 2.1. Individual-Level Factors

With respect to the overall uptake of PA and sports offerings, individual-level differences known to impact program participation and retention include socio-demographic [23,24], health-related [25,26], physical and psychological factors [27,28]. Accordingly, women, individuals with higher education and income levels, individuals with better objective and self-reported health, and those who are already actively involved in PA and sports are more likely to participate [29,30,31]. 

Studies of older adults’ specific requirements for PA interventions also revealed specific differences according to individual-level characteristics. For example, there is some evidence that particularly older individuals [31,32], as well as those with lower socioeconomic status (SES), and those who are overweight, or had poorer exercise habits in the past [24], are susceptible to non-participation. Other reasons described as barriers to participation are a lack of information, time and/or interest [33,34], mobility and travel issues/problems with (access to) transportation [34,35,36], health problems [34,36] or being already sufficiently active [33]. Paralleling participation, women, higher educated adults [26,37] and those of older age [25,26,37] are more likely to stay in programs. In addition, there are indications that men, in contrast to women, are more skeptical of engaging in group activities [38]. 

### 2.2. Ecological Factors

In line with social-ecological model approaches [20,39], there is a growing recognition of potentially relevant ecological factors influencing PA behavior in general, such as the physical and social environment [40]. In addition, evidence from systematic reviews suggests that ecological conditions affect participation in local sport offers and social activities of older people [3,41].

#### 2.2.1. Socioeconomic Composition of the Neighborhood

The importance of integrating the geographical context into health research is exemplified by a considerable number of studies demonstrating associations of residence in a deprived neighborhood with lower levels of PA [42,43] or sports participation rates [44], while suffering from a higher number of health problems and health care inequalities [45]. Despite being in greater need, older adults in deprived areas are reported to have lower interest in participating in community-based activities associated with financial costs (e.g., for transport) [46] or have limited access to public transport and have, thus, trouble reaching certain facilities (e.g., facilities that exercise classes are held in) [47]. In addition, a small number of studies identified a greater risk of attrition in PA programs occurring in individuals living in areas with low and medium socioeconomic status [23,48]. 

#### 2.2.2. Distance

Due to the aging of societies, access to, and living in close proximity to, services of daily needs and health-related facilities is becoming increasingly important [41]. Along with distances, the effort associated with a claim in the sense of commuting times and costs must be taken into account. This is of crucial relevance, as this group is generally facing more financial and physical limitations than others. 

Some studies have demonstrated the importance of accessibility and proximity of facilities for PA in general [38,49], or indicated inaccessibility or lack of transport as barriers for engagement in exercise offers [50]. Given that with increasing age, individuals become more susceptible to large distances [51], inadequate access may prevent them from participation [46]. A qualitative study has highlighted proximity as an important element facilitating participation and retention in PA intervention studies, stating that participants who lived far away from the study sites or who moved far away during the study period were difficult to retain in the study [52]. Access to research sites is also a main concern often reported by older adults [53]. 

Research on older adults’ participation in prevention classes found benefits of short distances to course locations [54,55] or, conversely, identified larger distances as a barrier, with decreased opportunities for usage and higher costs for transport [56]. Thus, a preference has been shown for classes that are in close proximity to reduce travel time and costs [17]. Basche et al. gathered specific reasons explaining why access and transport are a problem in that age group, e.g., worries about driving in bad weather and about the amount of time which must be spent to get to the study center [57]. Distance between a participant’s home and the study site was also found to be a significant factor impacting enrolment and retention in a longitudinal aging study, with greater participation rates for closer distances and, in addition, higher dropout risk for increasing distances [58]. In a study by Farber et al., participation in daily or social activities was significantly reduced among older adults residing in car-dependent neighborhoods, e.g., those in the suburban or rural areas [59]. Consequently, a recent literature review revealed the implementation of studies close to the place of residence of the participants to be important for high retention rates [34]. 

However, the findings published to date are based on studies conducted in countries (such as the United States of America, with distance scales between 50 and 500 miles) that can neither be generalized nor transferred to other regions with different experiences depending on the social or cultural context. Regardless of the mode of transport, in Germany, 44% of daily trips are completed within ten minutes or less and around 70% take no longer than 20 min [60]. For European older adults, several studies found that women and those with a low income make fewer trips of shorter distances compared to younger populations, men, and those with higher earnings [61,62].

#### 2.2.3. Weather Conditions, Season and Holiday Periods

According to the evidence of systematic reviews, the weather is an environmental factor affecting individuals’ PA and mobility in day-to-day life [42,63]. Older adults are particularly more vulnerable to weather-related factors compared with younger age groups [64] and are more likely to change the way they go about their daily activities depending on the weather conditions [65]. Rainy, snowy and windy weather conditions, as well as conditions of extreme heat are assumed to prevent trips and cause daily activities to be postponed or avoided [65,66]. Moreover, precipitation can affect both active transportation, as well as motorized travel (car and public transport) [57,67]. 

A study on older, urban women and their attendance in an exercise class found adverse weather conditions, such as heat, wind chill, and snowfall in the hours before the class to be associated with a lower likelihood of attendance [68]. Furthermore, the authors noted a negative impact of the daily number of sunlight hours, assuming the class attendance to be substituted by other outdoor activities in warmer seasons [68]. These findings are supported by a longitudinal study in older US-American adults on the effect of weather indicating cold and snowy weather to be highly associated with a decreased exercise class attendance rate [69]. In addition, in a Canadian qualitative study, 30% of participants reported harsh weather conditions, especially winter weather, as a barrier to participation in exercise classes [70]. Nevertheless, similar to the results regarding the distances, the evidence of the cited studies is not transferable to the German context due to different climatic conditions.

Furthermore, instructor- or design-related factors and organizational determinants (such as program characteristics, time, location/setting) have been previously identified as playing a role when it comes to retention of participants [17,31,71]. 

Finally, seasonality might affect day-to-day planning and PA behaviors through weather variations as well as individual arrangements of leisure time. For example, public holidays or school breaks may encourage grandparents to take care of their grandchildren. Accordingly, a study investigating older adults’ decisions to stop participation in a resistance training program reported holidays as one of the three main contributing factors for withdrawal [72].

In sum, research, particularly regarding ecological determinants of older adults’ participation in and dropout from PA interventions, is still limited and there are almost no findings for German settings. This is a major shortcoming, because older adults have a higher prevalence of co-morbidities and disabilities than the general population, which is generally associated with lower adherence [73]. Thus, three study aims are formulated for this article:

(1) To determine whether the findings regarding individual-level factors associated with reduced participation and retention available thus far can be supported by the present study, which enrolled older German adults.
(2) To identify ecological predictors and program-related determinants of overall participation and retention. Consideration will be given to the socioeconomic composition and setting (urban vs. suburban) of the participant’s neighborhood, as well as the proximity to intervention sites.
(3) To determine associations of concise weather exposures and (public) holidays with weekly attendance rates in scheduled exercise classes.

## 3. Materials and Methods

### 3.1. Data and Procedures

#### 3.1.1. Intervention Program

In this study, we used data originating from a community-based intervention trial conducted from May 2016 to November 2017 in the Bremen-Oldenburg metropolitan region in Germany. The study is part of the larger research network “Physical Activity and Health Equity: Primary Prevention for Healthy Ageing” (AEQUIPA) [74]. A detailed description of the study design, including inclusion and exclusion criteria, recruitment and randomization strategies and the intervention has been reported elsewhere [75,76]. Briefly, older adults, aged 65 to 75 years, living independently (without assisted living), drawn from the residents registration office (RRO) from five communities representing areas of low community readiness regarding PA programs for older adults [77], were invited to participate in a three-month randomized controlled PA trial via mail. 

During the study, the inclusion criterion of age was slightly expanded from 65 to 75 years to 60 to 80 years to allow for an inclusion of eligible partners or spouses of potential participants of a different age in the study. The size of the sample randomly selected in respective communities was proportional to the resident target population and was balanced by gender (1:1). The sample size calculation is described in detail in the published study protocol [76]. Reminders were sent out in cases of no response after two weeks. In addition to this main recruitment strategy, the study was also publicized in local newspaper articles with the option to call up the research team directly. Individuals were excluded from the study if they had already planned vacation during the intervention period, had certain medical conditions, severe health impairments, or had no PC/internet access. 

Participants were randomly assigned to either a web-based intervention with subjective PA self-monitoring (IG1), a web-based intervention with subjective and objective PA self-monitoring (IG2) or a waitlist control group (CG) [76]. Participants in the IGs completed an identical 10-week (week two to eleven) program engaging in weekly on-site trainer-lead PA group sessions for 90 min. They were encouraged to continue exercising at home, using web-based program material, and to monitor their PA with a web-based PA diary (both IGs) and an activity-tracker (only IG2). The CG was offered the intervention of IG1 after completion of the follow-up assessment, but without weekly group meetings. Study participants received questionnaires at the study sites at baseline (week one) and follow-up assessments (week twelve) and were asked to return them within one week via mail.

#### 3.1.2. Ecological Data

To evaluate the neighborhood level context, data for the administrative units were derived from the statistical offices of Bremen and the state of Lower Saxony, Germany. To measure the impact of proximity, the points of interest (POIs), such as residential addresses of participants, as well as study sites (where baseline and follow up assessments took place in week one and twelve) and intervention sites (with onsite classes in week two to eleven) were geocoded and, in addition, assigned to administrative units (districts). The roads data (linear features such as roads and footpaths) were extracted from Open Street Maps (OSM) via QGIS 3.2.3 (QGIS Development Team), OSM Plugin (Quick OSM, Version 1.5, QGIS Development Team). Daily accurate local weather data were recorded from publicly accessible databases maintained by the German Weather Service/Climate Data Center (CDC) [78]. In addition, national public holidays and local school breaks for the years 2016 and 2017 in Bremen and Lower Saxony (Germany) were retrieved from the website schulferien.org [79] in September 2021. Table 1 shows the types of data resources used.

### 3.2. Measures

#### 3.2.1. Primary Outcomes

For the analysis, the primary outcomes were the study’s response, participation and dropout rates. Response rate was defined as the number of responders in relation to the number of invited individuals. Participation rate was defined as the number of participants, including volunteers, who were included in the study after screening for eligibility in relation to the number of invited ones. Dropout rate was defined as the number of participants indicating that they were not willing or able to continue the program, in proportion to those who joined the intervention. In addition, attendance rates in weekly onsite classes, recorded by the respective assigned leaders, were calculated.

#### 3.2.2. Potential Explanatory Variables

##### District Level Socio Economic Status

For a district assignment and classification regarding urban and suburban neighborhoods, the participants’ zip codes were used. Because the use of established composition indices for assessing regional socioeconomic deprivation hardly revealed any heterogeneity in our data and that they are, in addition, prone to prevent the detection of differential correlations [80], we used data on the number (proportion) of welfare recipients from the welfare statistics as a proxy. Data were divided into quartiles scaled to the distribution of all administrative units in the study region with the first quartile (Q1) reflecting lowest and the fourth quartile (Q5) reflecting highest neighborhood SES.

##### Proximities to Interventions Sites

**Figure 1:**
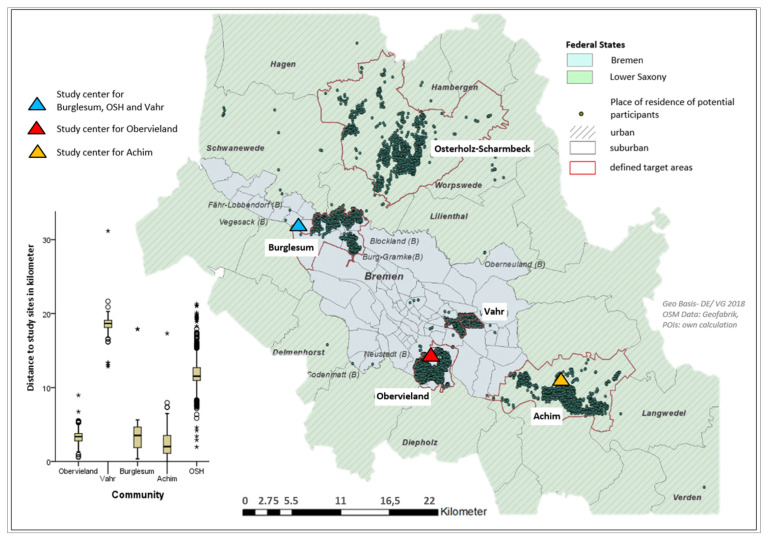
Invited participants in the recruitment sample by target area and communities, and distance to the study centers (where baseline and follow-up assessments took place in week one and twelve). Note: OSH: Osterholz-Scharmbeck, * individual outliers within the Boxplot.

**Figure 2:**
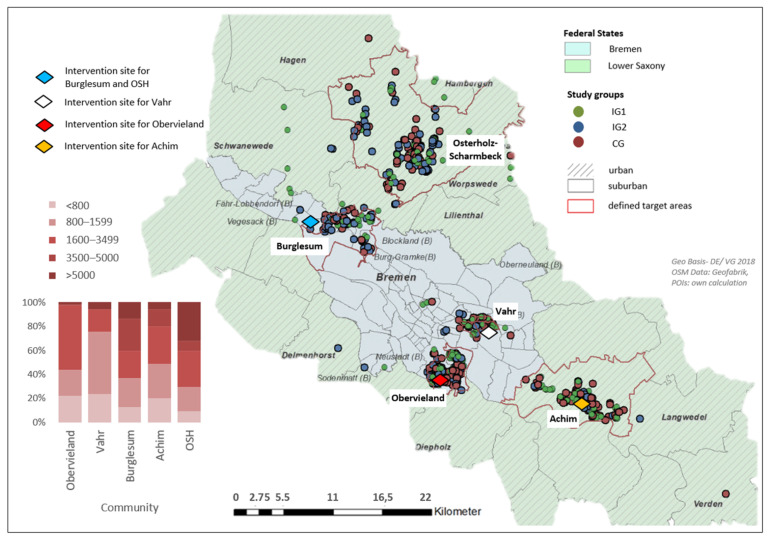
Participants of study (*n* = 589) by target area, communities and distance to the intervention sites (where onsite classes took place from week two to eleven). Note: OSH: Osterholz-Scharmbeck.

The network analysis tool in QGIS 3.2.3. (QGIS Development Team) was used to calculate the distance between respondents’ georeferenced home locations and georeferenced study and intervention sites. The latter varied depending on the community (Figure 1 and Figure 2). To ensure a routable network, all POIs needed to be snapped properly to the network, something which was not the case in the initial data set. Therefore, they were matched via QGIS snapping tools to the closest vertices (nodes, junctions, road bends) of the street network [81]. Using the street network data, proximities based on the shortest possible path were measured. The most appropriate geographic scales regarding older adults’ PA and mobility in health and planning research are still unknown [82]. Therefore, the choice of cut-offs for five distance categories started with thresholds for walkable distances commonly used in built environment and health research, which represent an approximate walk time between (or up to) 10 min (800 m) and 20 min (1600 m) [83,84]. 

As participants’ geocoded homes were blurred for data protection reasons following a method described elsewhere [85], the real coordinates are slightly shifted. Following the methodology, home coordinates in low populated areas (based on the number of inhabitants) are moved by a larger factor than in densely populated areas, which particularly affects participant residences located in more distant, suburban areas. As the distance categories become broader in the upward direction (greater proximities), the study centers (not the participants’ blurred home addresses) were set as the starting point for the distance measures to compensate for the potential shift bias term.

##### Weather, Season, Public and School Holidays 

The weather data (including total rainfall, mean temperature, and wind speed) were selected and classified according to the threshold of significant weather exposures defined by the German Weather Service (GWS) [86]. As the study continuously recruited participants in various recruitment waves and participants were assigned to different groups, the weather on the days of the group sessions within each of the 10 weeks of the onsite classes differed between the groups. We examined whether significant weather for temperature (in degree Celsius above 30 or below zero), rainfall (>15 l/m^2^ within an hour) and/or wind speed (>7, which is 60 km/h) was present on the days under consideration, using binary dummy variables (YES = 1/NO = 0). 

To account for the effect of season, the variables winter (December through February), spring (March through May), summer (June through August), and autumn (September through November) were coded.

Public holidays in Germany (e.g., Labor Day, German Unity Day, Reformation Day, Ascension Day, Whit Monday, and Easter Monday, etc.) were considered if a given class day was the day before, the day of, or the day after the holiday. Additionally, information was collected on whether the respective intervention period occurred during school breaks.

##### Individual-Level Characteristics

Of the individuals who were included in the study, and for whom a dropout analysis was calculated, age and gender were obtained during the initial telephone interview, and data on education level, employment status, household income and self-rated health (subjective) status were collected during the baseline assessment via questionnaire. Level of education was coded following the 2011 version of the International Standard of Education (ISCED), e.g., individuals with a higher educational status received a higher score (range 1–8) [87]. This was further dichotomized into low/moderate (ISCED 1 to 4) and high (ISCED 5 to 8) levels of education. Employment status was assessed using one item from a questionnaire for assessing demographic and socio-structural characteristics [88]. Household income was assessed using items of the German Health Interview and Examination Survey for Adults [89]. Need-weighted income per capita was derived considering the monthly household income and the number of individuals living in the household according to the German Microcensus [90]. The variable was then tertiled into low, middle, and high household income. Health status was assessed by asking older adults to rate their general health status using an item from the Short Form-36 Health Survey [91].

### 3.3. Statistical Analysis

Where the data were available, the analysis explored differences in individual and ecological participant characteristics between responders, non-responders, participants finally included in the study (according to the eligibility criteria) where available, as well as dropouts and completers. Descriptive statistics were summarized as number of cases with percentages for categorical variables, as well as the mean and standard deviation, as appropriate. In order to assess significant group differences between dropouts and completers, all categorical variables were compared by using the Pearson chi-square and Cramer’s V tests. Attendance in onsite classes was calculated as the ratio of the number of actually present participants to the number of expected participants, considering dropouts. Because we did not track individual attendance in classes, this analysis could only be performed at the group level.

An appropriate method to assess predictive values for dropout is survival and Cox-regression analysis [10], which includes a time-to-event variable. Ecological, as well as person-based data described above, served as basis for a descriptive analysis plotted as Kaplan–Meier curves to visualize the time until dropout by predictor variables. The equality of the survivor functions was tested with a log-rank test (*p* < 0.05). Survival analysis was performed to examine predictors of dropout among participants who started the intervention. The total number of weeks (1–12) was used as time variable for the event (dropout). Cox Proportional Hazards models were calculated to investigate the effects of potential predictors on dropout. 

Age, gender, community, neighborhood setting (urban/suburban), intervention group, level of education, employment status, household income, subjective health, district level SES, distance to intervention sites and recruitment characteristics (contacted vs. volunteering persons) were first examined within a univariate (unadjusted) Cox proportional hazard regression. Predictors that showed noticeable differences regarding the relative hazard for dropout (hazard ratio, HR) were selected for inclusion in a multivariate (adjusted) regression model, even if they did not confirm to be significant (*p* < 0.05). The latter was tested in a stepwise backward procedure by sequentially excluding variables with *p*-values ≥ 0.05 based on the likelihood ratio statistics. Finally, variables that significantly (*p* < 0.05) predicted dropout were kept in the final model. HRs and their 95% confidence intervals were calculated, and the Wald test was used for model testing. 

Only participants with full data sets of all covariates to be studied (*n* = 539) were included in multiple regression models. Statistical analyses were performed using SPSS, IBM version 25 for Windows [92]. All spatial analyses described above were completed using QGIS, Version 3.2.3. for Windows (QGIS Development Team).

## 4. Results

### 4.1. Results Regarding Participant Enrollment 

**Figure 3:**
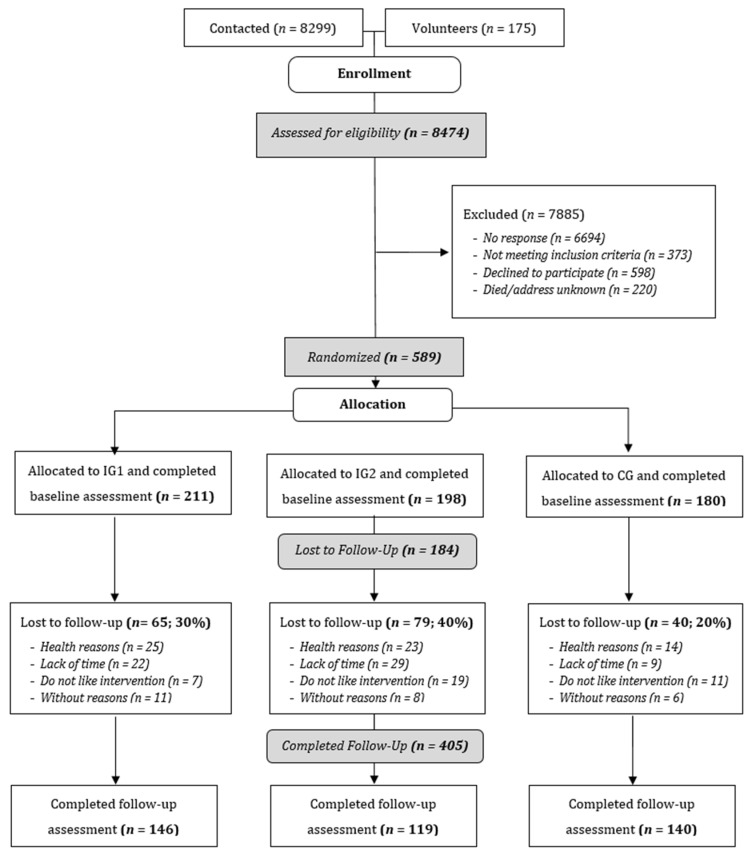
Flow chart. Note: CG: waitlist control group, IG1: web-based intervention with subjective PA self-monitoring, IG2: web-based intervention with subjective and objective PA self-monitoring.

A random sample of *N* = 8299 older adults was invited to participate in the study via mail, with 6694 not responding to the invitation letter and 598 proactively declining to participate. Men responded slightly less than women (81% vs. 76.6% nonresponse). The total non-response rate was 80.7%. For 220 individuals, the reason for exclusion was “death/address unknown”. During the assessment for eligibility during the initial telephone interviews, an additional 373 potential participants were excluded for not meeting the inclusion criteria (see Figure 3). A total of 459 (77.9%) contacted older adults were included in the study. Additionally, of 175 people who reacted to the press release and decided to participate in the study, 130 were eligible for inclusion. Finally, 589 study participants were randomized to the study groups: IG1 (*n* = 211), IG2 (*n* = 198) and CG (*n* = 180). 

Table 2 presents the characteristics of non-participants (non-responders as well as responders and volunteers who were not eligible for the study) and participants. There was no substantial difference in gender and setting (urban/suburban) towards response. In total, the districts with very high community levels of SES had the largest number of non-responders (84.4%) based on the number of individuals contacted in that quartile in contrast to the lowest non-response rate from districts with the lowest SES (76.8% of individuals contacted). The non-response rate with respect to the different communities varied from 75.5% (Osterholz-Scharmbeck) to 82.3% (Vahr) of the contacted persons in the given community. Most of the included (eligible) participants came from the community Obervieland (28.7%) and the fewest from the community Vahr (14.4%). Further, the majority of participants were retired (77.1%), female (58%), with low or moderate educational status (52.3%), with a good health status (56.6%), with high household income (34.5%), and resided in districts with low level of SES (62.1%). 

### 4.2. Results Regarding Retention

Between the years 2016 and 2017, a total of 589 participants started the intervention. Finally, *n* = 405 participants (69%) completed the follow-up assessment after 12 weeks (IG1, *n* = 146; IG2, *n* = 119; CG, *n* = 140). IG2 displayed the greatest loss-to-follow up (Figure 3). Overall, the mean age of the included study participants was 71.4 years (SD = 3.3) with higher, but non-significant dropout rates among the youngest and oldest age groups, women, volunteers, those with a lower household income, and those with the shortest and highest distances to intervention sites. 

In total, the highest dropout rate appeared in those with higher SES at the district level (*p* < 0.05). There were significant (*p* < 0.01) variations between the different communities regarding the dropout rates (Table 2). 

In addition, this analysis revealed that lower educated older adults (*p* < 0.05), those who reported less good or poor health (*p* < 0.01), and those who lived in urban neighborhoods (*p* < 0.01) were more likely to drop out. Although the study participation occurred in the spring/summer for the vast majority of participants, proportionally fewer participants that started the program in the fall/winter dropped out (*p* < 0.01).

### 4.3. Survival Analysis and Stage of Dropout

**Figure 4:**
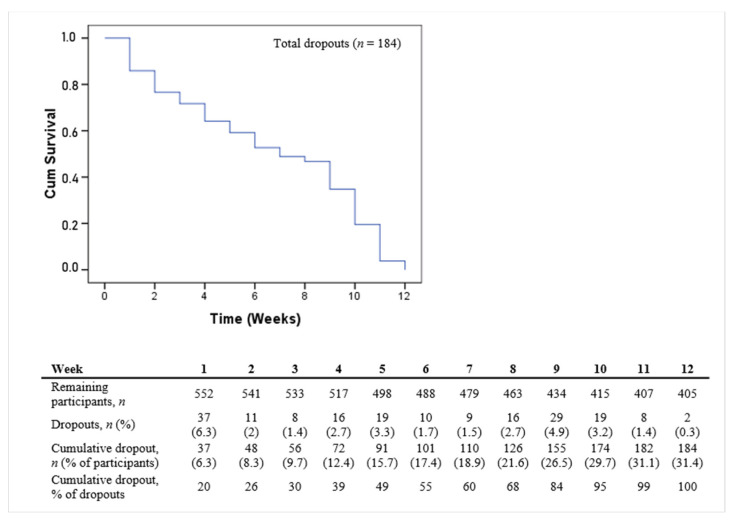
Time until dropout within 12 weeks for those who left the study (*n* = 184). Week 1: baseline assessment one week before start of the intervention, week 12: follow up assessment following the intervention, weeks 2–11: intervention program.

**Figure 5:**
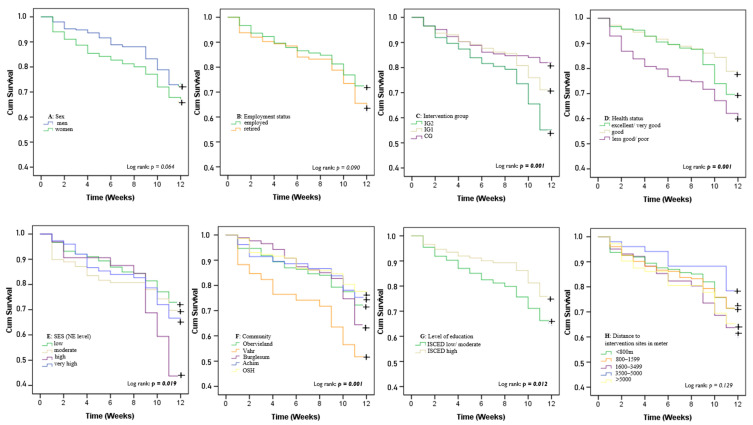
Survival plots for the time until dropout from the study comparing different variables (A = sex, B = employment status, C = intervention group, D = health status, E = SES, F = community, G =level of education, H = distance to intervention sites). Note: 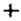 censored data = end of observation period (week twelve).

The Kaplan–Meier curves in Figure 4 and Figure 5 show the participants’ time until dropping out of the study. Overall dropout rates, starting with the baseline assessment (week 1), continuing with an introductory event (week 2), followed by ten weeks of the program, and ending with the follow-up assessment (week 12), indicate that between the fifth and sixth week, half of the dropout population had left the study, with the largest decrease occurring in the first week (Figure 5). Median survival time (the point at which 50% of the participants were still adherent) could also be directly read from the Kaplan–Meier curve (Figure 4). Of those who dropped out (*n* = 184), most of the attrition occurred within the first (*n* = 37, 6.3%) and the ninth week (*n* = 29, 4.9%).

**Table 2:** Individual and community level characteristics.

Characteristics	Contacted and Volunteers	Non-Responders *	Included Participants	Completed Study **	Dropouts **	*p*	V
***N*** **-Total (%)**	8474	6694	589 (7%)	405 (68.8)	184 (31.2)		
**Recruitment, *n* (%)**							
Contacted	8299	6694 (80.7)	459 (77.9)	324 (70.6)	135 (29.4)	0.072	0.074
Volunteers	175		130 (22.1)	81 (62.3)	49 (37.7)		
**Community *n* (%)**							
Burglesum (urban)	1085 (12.8)	855 (78.8)	87 (14.8)	55 (63.2)	32 (36.8)	**0.002**	0.172
Vahr (urban)	2300 (27.1)	1892 (82.3)	85 (14.4)	44 (51.8)	41 (48.2)		
Obervieland (urban)	2257 (26.6)	1734 (76.8)	169 (28.7)	121 (71.6)	48 (28.4)		
OSH (suburban)	1457 (17.2)	1100 (75.5)	143 (24.3)	108 (75.5)	35 (24.5)		
Achim (suburban)	1375 (16.2)	1109 (80.7)	105 (17.8)	77 (73.3)	28 (26.7)		
**Neighborhood Settings, *n* (%)**							
Urban	5642 (66.6)	4481 (79.4)	341 (57.9)	220 (64.5)	121 (35.5)	**0.009**	0.107
Suburban	2832 (33.4)	2209 (78.4)	248 (42.1)	185 (74.6)	63 (25.4)		
Missing		4					
**Age in years, mean (*SD*)**			71.4 (3.3)	71.3 (3.2)	71.6 (3.5)		
**Age groups, *n* (%)**							
60–64			5 (0.8)	3 (60)	2 (40.0)	0.552	0.060
65–69			196 (33.3)	136 (69.4)	60 (30.6)		
70–74			305 (51.8)	215 (70.5)	90 (29.5)		
75–80			80 (13.6)	50 (62.5)	30 (37.5)		
Missing			3 (0.5)	1 (33.3)	2 (66.7)		
**Gender, *n* (%)**							
Male	4146 (48.9)	3376 (81.4)	251 (42)	182 (72.5)	69 (27.5)	0.096	0.069
Female	4328 (51.1)	3314 (76.6)	336 (58)	223 (66.1)	114 (33.9)		
**Season, *n* (%)**							
Fall/winter		1872 (28)	165 (28)	135 (81.8)	30 (18.2)	**0.001**	0.176
Spring/summer		4822 (72)	424 (72)	270 (63.7)	154 (36.3)		
**Level of education, *n* (%)**							
ISCED low, moderate			308 (52.3)	201 (65.3)	107 (34.7)	**0.019**	0.098
ISCED high			261 (44.3)	194 (74.3)	67 (25.7)		
Missing			20 (3.4)	10 (50)	10 (50)		
**Employment status, *n* (%)**							
Employed or retired but working			113 (19.2)	71 (62.8)	42 (37.2)	0.070	0.076
Retired or other			454 (77.1)	325 (71.6)	129 (28.4)		
Missing			22 (3.7)	9 (41)	13 (59)		
**Household income, *n* (%)**							
Low			164 (27.8)	109 (66.5)	55 (33.5)	0.299	0.067
Middle			168 (28.5)	118 (70.2)	50 (29.8)		
High			203 (34.5)	150 (73.9)	53 (26.1)		
Missing			54 (9.2)	28 (51.9)	26 (48.1)		
**Subjective health status *n* (%)**							
Excellent or very good			144 (24.5)	116 (80.6)	28 (19.4)	**0.001**	0.188
Good			333 (56.6)	234 (70.3)	99 (29.7)		
Less good or poor			87 (14.7)	46 (52.9)	41 (41.1)		
Missing			25 (4.2)	9 (39)	16 (64)		
**District level SES, *n* (%)**							
First quartile (low)	3779 (44.6)	2904 (76.8)	366 (62.1)	262 (71.6)	104 (28.4)	**0.011**	0.138
Second quartile (moderate)	1911 (22.6)	1509 (79)	109 (18.5)	76 (69.7)	33 (30.3)		
Third quartile (high)	625 (7.4)	482 (77.1)	32 (5.4)	14 (43.8)	18 (56.3)		
Fourth quartile (very high)	2102 (24.8)	1774 (84.4)	75 (12.7)	49 (65.3)	26 (34.7)		
Missing	57 (0.7)	25 (43.8)	7 (1.2)	4 (57.1)	3 (42.9)		
**Distance to intervention sites in meters, *n* (%)**							
<800 (very low)			102 (17.3)	62 (60.8)	40 (39.2)	0.118	0.112
800–1599 (low)			161 (27.3)	115 (71.4)	46 (28.6)		
1600–3499 (moderate)			203 (34.5)	143 (70.4)	60 (29.6)		
3500–5000 (high)			51 (8.7)	40 (78.4)	11 (21.6)		
>5000 (very high)			72 (12.2)	45 (62.5)	27 (37.5)		
Missing							

Note: * % from contacted in that category, ** % from included in the category, *p* = chi square-test group differences (completer/dropouts), with those in bold type indicating significant values, V = effect size by Cramer’s V, ISCED: International Standard of Education, OSH: Osterholz-Scharmbeck, SES: socioeconomic status.

In the group comparisons studied regarding time until dropout, men stayed longer in the intervention than women and were more likely to stay during the first week (Figure 5A). In addition, there were some significant differences according to the log-rank test. For example, participants in the control group remained in the intervention longer than those in IG1 and particularly IG2 (*p* < 0.01) (Figure 5C). Those who reported less good health (*p* < 0.01) (Figure 5D), living in districts with a high level of SES (*p* < 0.05) (Figure 5E) or reported a low or moderate level of education (*p* < 0.05) (Figure 5G) left the study the earliest. Figure 5F illustrates a high dropout in the first week among those from the community Vahr, something that can probably be attributed to the large distance to the study center in which the baseline assessments were performed during the first week (Figure 1).

This is supported by the Cox Proportional Hazards analysis presented in Table 3, indicating these factors as significant predictors (*p* < 0.05) in the univariate model. The HR of dropping out was 2.1 times higher for participants living in districts with high levels of SES compared with the HR for those in low SES districts (HR = 2.143, 95% CI 1.299–3.536, *p* < 0.01). In addition, those who were randomized to IG2 were significantly more likely to drop out (HR = 2.057, 95% CI 1.406–3.009, *p* < 0.01). Some factors significantly decreased the HR of dropping out, such as being highly educated (−32%, *p* < 0.05), living in suburban areas (−32%, *p* < 0.05%), living in high distances to intervention sites (3500–5000 m) as compared with the shortest distance (−30%, *p* < 0.05). However, the results regarding the distances do not indicate a trend in the sense of increasing or decreasing dropout rates with increasing or decreasing distances.

When performing multivariate regression, five models were calculated using backward stepwise selection. The first model (step 1) included all predictors that showed noticeable differences in the HRs in the univariate model, such as community, intervention group, level of education, subjective health status, employment status, district level SES, and distance to study sites. The variable “Neighborhood setting” was taken out of the multivariate model due to its mathematical correlation (sum of) with the factor “Community”. A correlation heat map of relevant variables is provided in the Supplementary in Figure S1. Contrary to the univariate model, the influence of the ecological factors (district level SES and distance to intervention) sites was no longer significant when controlling for the other variables in the analysis. On the other hand, self-reported health appeared to be a relevant determinant in the multivariate model (*p* < 0.01).

After stepwise exclusion of all non-significant (*p* ≥ 0.05) factors, four variables remained in the final model (step 5), namely community (*p* < 0.05), intervention group (*p* < 0.01), level of education (*p* < 0.05) and subjective health status (*p* < 0.01) (see Table 3). Compared with the basic model, when other factors (district level SES, gender, distance to study sites and employment status) were removed, the final model revealed no severe changes with respect to the predictors, except for marginal improvements in the model significance levels. In conclusion, being randomized to CG was a significant predictor for a low HR of dropping out (*p* <0.05). For IG2, the HR to leave the program before study completion was 2.7 times higher compared with CG (HR = 2.666, 95% CI 1.737–4.093, *p* < 0.01). The greatest risk of dropout occurred for participants from the community Vahr (HR = 1.803 95% CI 1.155–2.815, *p* < 0.01). Being highly educated reduced the risk of dropout by 33% compared with those with low or moderate educational levels (HR = 0.674, 95%, CI 0.448–0.931, *p* < 0.1). Furthermore, self-reported less good or poor health increased the risk of leaving the study before completion by about 2.6 times compared with those that reported excellent or very good health (HR = 2.644, 95% CI 1.590–4.396), *p* < 0.01).

### 4.4. Attendance in Weekly Onsite Classes 

**Figure 6:**
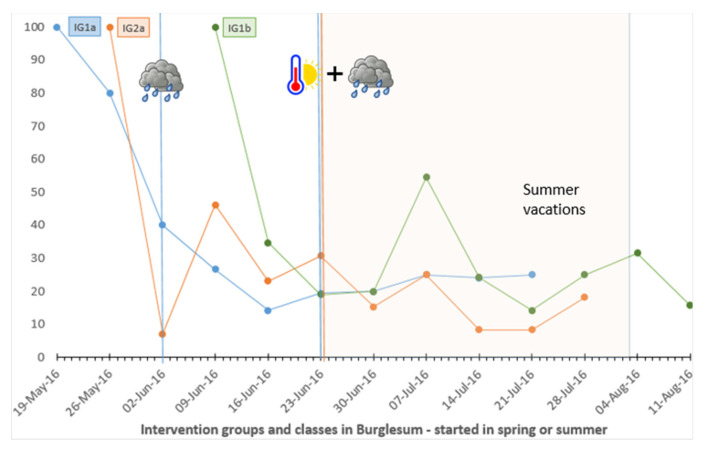
Weather expositions and holiday periods by days of class meeting and rate of expected attenders in different groups in the community Burglesum. Note: 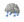
rainfall > 15 l/m2, 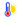 temperature > 30. IG1a: web-based intervention with subjective PA self-monitoring, first group, IG1b: web-based intervention with subjective PA self-monitoring, second group, IG2a: web-based intervention with subjective and objective PA self-monitoring, first (only) group.

Figure 6 presents the attendance rates of 10 weekly on-site classes (week two to 11 of the study) for three different groups in the community of Burglesum based on the expected number of attenders (those who had not dropped out of the study before the next class). Those who left the study before the first or after the 10th class were not included. Considering all communities, on average, the attendance rate decreased by almost one half (46.4%) in the second week compared to the first. The level of attendance varied between the communities (Supplementary Figure S2).

The analysis of the extracted weather data yielded only a few days with significant weather exposures, for the period under consideration, of which the majority were wind exposures (affecting 19 class days within 11 different groups) (Supplementary Table S1). Nevertheless, the descriptive results, including holiday periods, revealed no clear trend regarding the association with attendance (further illustrations can be found in the Supplementary Figure S3).

## 5. Discussion

This article addresses the need to increase participation and retention in PA interventions, including attendance in weekly onsite PA classes among older adults, taking into account a range of potentially influencing factors based on a social ecological approach. Although the impact of ecological factors has been formulated as the initial key interest in response to the research gap, such an analysis would fail without considering individual and organizational factors. The findings from this study are discussed below in the context of existing evidence.

### 5.1. Principal Findings

In general, participation rates in our study were comparable to findings of a systematic review (7% versus 9%) (86). Seven percent (*n* = 589) of the contacted and voluntarily registered older adults (*n* = 8474) participated in the study and were randomly allocated to one of two intervention groups or a delayed control group. In total, retention in our study was somewhat lower than in those included in a previous systematic review (69% versus 80%) [84]. Nevertheless, varying attrition rates have been reported in the past, ranging from 22% to 76% for exercise interventions [93] and 0% to 62% for web-based PA interventions [94], which support previous findings characterizing e-health interventions as those with high dropout rates [10,95].

### 5.2. Individual-Level Factors Associated with Participation and Dropout

In line with previous research [96], we found that individuals who were older and not employed were more likely to participate and also to complete the study. This could be because adults who are not working have greater availability and flexibility regarding scheduling than younger and non-working ones [38]. Similar to former research, we found men to be less likely to participate [29,30,94], but for those who did, there were indications (*p* < 0.1) that they appeared to remain in the study longer than women [12]. Regarding e-health interventions, some research suggests that men generally have more positive attitudes and self-efficacy expectations towards new technology than women and, thus, are more attracted by interventions based on technical devices [97]. In addition, our results support those of other studies revealing that being less healthy is linked to attrition [23,26,37]. In contrast to existing studies, we found a slightly higher proportion of low or moderately educated participants in our sample but, as identified by earlier research [26,37], proportionally higher educated participants completed the intervention. This may reflect the fact that awareness and understanding of the role of PA for health issues affects adherence to interventions.

Our analysis did not reveal age and household income as significant (<0.05) predictors for dropout from the study, while other studies did [25,26,37]. However, the results on household income are difficult to compare, as we used the need-weighted income per capita for Germany as a basis, which is not necessarily transferable to other countries.

### 5.3. Ecological Predictors Associated with Response, Participation and Dropout

In line with socio-ecological approaches, this study considered a number of factors that might contribute to a better understanding of (non-)participation behavior by looking beyond the individual level. For example, those of the physical and social environment, as well as those at the organizational level.

We found the largest non-response among individuals living in districts with very high community SES levels. At the same time, we found low district level SES to be significantly associated with remaining in the study, while other studies identified the highest dropout rates among participants living in areas with a lower SES [23]. Another finding of our study was that participants who lived in suburban areas were less likely to leave the study in contrast to those residing in urban areas. It can be assumed that these participants tend to commute by car, as former studies suggest [98,99], and accessibility therefore is less of a barrier. Another explanation, supported by a recent systematic review, could be the limited supply of PA programs and classes in suburban areas and therefore the need to take advantage of existing ones, even with greater individual efforts [50]. However, this contradicts findings of a Canadian study identifying reduced participation in activities among older adults residing in car-dependent neighborhoods, such as suburbs [59]. One has to take in mind, that the typical environment looks different in Canada and Germany with the current study conducted in a more nearby assembly which might ease the retainment in the study. 

Regarding accessibility, there were indications for the impact of proximity in our study when considering a community with a large distance to the study site (where the baseline assessment took place) and a high dropout rate during the week of the assessment. In addition, we found that participants with high proximities to study sites remained in the program longer compared with those with the smaller and the greatest distances. This is not consistent with other study findings emphasizing the benefit of close proximities [54,55]. These differences may be seen as an effect of the individual effort required to get to the site [38], not only the mere distance. 

In our study, participation and dropout rates varied depending on the community that participants resided in. However, it is important to note that the differences are not solely determined by the ecological factors studied. Our findings and the remaining predictors in the final model of the Cox regression could be an indication for the high impact of differing program factors, which became already obvious in previous studies [71,100]. These may include the perception of and experience with intervention facilities, time slots (for the study assessment and/or the intervention classes), as well as the personality and professionalism of the assigned leaders of the onsite classes, which were not systematically assessed in our study, but in previous research [16,17], and which may have differed by community in our study. 

### 5.4. Factors Associated with Recruitment and Study Design 

Recruitment of volunteers has long been a concern of research, as volunteers often differ from the general population in ways that are directly related to the study outcomes, as well as to participation and retention [23,29]. As recruiting via media and events was only a secondary strategy for this study, the proportion of individuals calling the team up to participate in the study was only 30%. Although individuals who contacted the study team themselves to participate are assumed to be more motivated and committed to the study, which led to lower attrition rates in the past [10,23], we found no significant differences for volunteers leaving the study before the end compared to contacted individuals. 

In addition to individual and ecological factors, characteristics of the intervention itself may affect dropout [96]. According to Eysenbach, attrition might also be the result of a wrong or mismatched intervention treatment [10]. In our study, randomization to intervention groups as a factor related to the study design appeared to be a significant predictor for retention. We identified higher dropout rates in the IGs than in the CG. This difference may be explained by the fact that participants in waitlisted groups are typically more motivated to stay in the study, due to the expectation of receiving the program afterwards [10]. Furthermore, they are probably less likely to discontinue due to time constraints, because they do not receive appointments interfering with that. In addition, the discrepancy between IG1 and IG2 regarding dropout supports technology-based causes (such as lack of technical competences or experiences) identified earlier [76,101].

### 5.5. Impact of Season, Weather Conditions and Holiday Periods

In line with a previous review identifying certain seasons as barriers for PA [63], this factor appeared to have an impact on participation in our study. The higher dropout rates in our study that occurred in summer (versus winter) may support findings from previous research, indicating that individuals tend to use their spare time for more personal outdoor activities during the warm and sunny months [68]. However, this is contradicted by findings from qualitative studies with participants stating that they prefer courses in summer months as they do not occur or finish after dark [102]. Consequently, there should be a varying offer covering these different needs at best.

However, we found no clear evidence regarding poor and extreme weather to transfer the findings of that review to the results obtained for attendance in onsite classes in our study. One reason could be that the cutoffs for the weather expositions were somewhat inappropriate and that a differing classification would have yielded other results. In our study, the public holidays and school breaks revealed no obvious effects on class attendance. Probably, PA interventions like this might act as a substitute for the more limited access to public sports clubs and the reduced number of exercise classes during vacation periods.

Similar to our study, former research found ecological factors to explain less of the variations compared to individual-level factors [103], which, however, does not mean that they require less attention during intervention planning. Regarding retention, it is conceivable that for those who once decided to participate, ecological factors, such as distances, no longer play a role. Rather, it appears to be that individual-level factors, such as demands of the participants, or details of the program itself, which have been suggested to be related to early dropout [38,71,100], are more important. Research on the socioemotional selectivity theory has found emotional gratifications and support to be particularly important for older adults [104]. This might reflect the impact of the group composition itself as well as that of the group leadership for the attendance behavior towards the onsite classes. 

Because emotional selection processes also seem to increase voluntary activities [105], future research might already take advantage of such findings in the early recruiting phase. Consideration should also be given as to how to appeal to different subgroups of the older adult population, such as those with low levels of education or low subjective health. This study found that there have been difficulties in engaging men in organized PA sessions, which confirms findings of previous research and have led to the development of older men only activities [102]. Within this study, there were greater numbers of finishers in groups with low requirements for technical skills. These challenges, as well as that of motivating the uninterested individuals, will need to be addressed in a revision of the image and design of the intervention in the future.

### 5.6. Strengths and Limitations

This study is among the first to examine ecological factors, such as proximities, weather conditions, and socioeconomic status at the neighborhood level, in a German context to explain inequalities regarding participation and retention in a PA intervention. To the best of our knowledge, there is no other study to date that has investigated study dropout using GIS based network analysis. A further strength is the variety of analyzed indicators. In particular, we used both geocoded data and open data, such as street network data and weather data, and demonstrated how GIS analysis could be used to evaluate access. The detailed documentation of study dropout, as well as the sufficient number of study participants, enabled us to perform survival analyses for various subgroups.

However, some limitations need to be considered. First, we enrolled individuals with minimal comorbidities and rather independent individuals regarding their activities of daily living, which does not adequately reflect reality. In addition, the composition of our study sample suggests that predominantly active older adults are particularly attracted to PA programs, which is a well-known problem in recruiting participants for health promotion research, suggesting a selection bias. Appropriate strategies are still lacking. Furthermore, some of the respondents had to be excluded from the study because they did not meet the inclusion criterion of owning a PC or did not have access to the internet. This may have led to the exclusion or insufficient recruitment of socioeconomically disadvantaged older adults and may have finally reduced the representativeness of the study sample. 

Second, as we did not track individual attendance in group meetings, this part of the analysis could only be performed at a group level. Third, due to missing values for age in the contacted sample (about 50%), no conclusions can be drawn regarding age as a potential correlate of response. Fourth, for this quantitative analysis, we disregarded the self-reported reasons for withdrawal. This was partly because many participants did not provide any specific information, and because factors might overlap. Finally, the modifiable areal unit problem (MAUP) is a source of bias in research of spatial phenomena, therefore also in this study. It states that results are affected by the shape and scale of the aggregation unit (e.g., districts) [106].

## 6. Conclusions

Identifying different predictors of older adults’ participation and retention can help improve future interventions for the promotion of healthy aging. Our results suggest that a key element is knowledge of the composition and needs of the targeted group. As older adults are a very heterogeneous group, tailoring PA interventions more accurately according to individual level determinants (including perceived health conditions and levels of education) may prevent high dropout. 

Because program-related characteristics also appear to be of great importance, they might also already be taken into account during the planning stage. 

However, further research is needed to obtain a robust conclusion on the influence of environmental factors on participation and retention in health interventions for older adults, such as programs for PA promotion. Improvements in the quality of the existing data and the measurement methods (e.g., cut offs for proximities and weather expositions) might lead to clearer results in the future.

## Figures and Tables

**Table 1:** Assessed characteristics and data sources.

	Response		Dropout from the Study and Attendance in Weekly Onsite Classes	
*N*	*Characteristics*	*Data Source*	*Characteristics*	*Data Source*
Individual level	Age	Registration office	Age	Telephone interviews
	Gender	Registration office	Gender	Telephone interviews
	Residential address	Registration office	Residential address	Registration office
			Level of education	Questionnaire
			Employment status	Questionnaire
			Household income	Questionnaire
			Perceived health	Questionnaire
Ecological level	Neighborhood SES	Statistical offices ^1^	Neighborhood SES	Statistical offices ^1^
	Proximity	Geocoded home addresses	Proximity	Geocoded home addresses
		Geocoded study center		Geocoded intervention sites
		OSM street network data		OSM street network data
			Weather ^2^	GWS/CDC
			Season	Study Data
			Public/school holidays ^2^	schulferien.org

Note: ^1^ of Bremen and Lower Saxony, ^2^ only used to evaluate participation in the ten weekly onsite classes, CDC: Climate Data Center, GWS: German Weather Service, SES: socioeconomic status, OSM: Open Street Maps.

**Table 3:** Results of univariate and multivariate Cox Regression (*N* = 539). Notes: * Tested in a stepwise backward procedure by sequentially excluding variables with *p*-values ≥ 0.05, ** Removed from multivariate models due to high correlation with the community variable (see Supplementary Figure S1). Bold marks indicate significant results.

Characteristics	Univariate HR (SE), 95% CI	*p*	Multivariate HR Beginning Model (SE), 95% CI	*p*	Multivariate HR Final Model * (SE), 95% CI	*p*
**Community**						
Obervieland (urban)	Reference	**0.001**	Reference	**0.011**	Reference	**0.003**
Vahr (urban)	1.987 (0.213), 1.309–3.015	**0.001**	1.878 (0.253), 1.144–3.080	**0.013**	1.803 (0.227), 1.155–2.815	**0.009**
Burglesum (urban)	1.278 (0.228), 0.817–1.998	0.283	1.258 (0.282), 0.724–2.187	0.415	1.356 (0.252), 0.828–2.221	0.226
Achim (suburban)	0.923 (0.238), 0.579–1.407	0.735	0.671 (0.308), 0.367–1.227	0.195	0.777 (0.265), 0.426–1.306	0.341
OSH (suburban)	0.837 (0.222), 0.541–1.293	0.422	0.663 (0.295), 0.372–1.120	0.163	0.816 (0.235), 0.515–1.295	0.388
**Intervention Group**						
CG	reference	**0.001**	reference	**0.001**	reference	**0.001**
IG1	1.437 (0.201), 0.696–2.130	0.072	1.770 (0.232), 1.123–2.790	**0.014**	1.149 (0.229), 1.149–2.822	**0.010**
IG2	2.057 (0.194), 1.406–3.009	**0.001**	2.767 (0.223), 1.786–4.286	**0.001**	2.666 (0.219), 1.737–4.093	**0.001**
**Level of education**						
Low/moderate	reference		reference		reference	
High	0.683 (0.156), 0.503–0.927	**0.014**	0.693 (0.171), 0.495–0.969	**0.032**	0.674 (0.165), 0.488–0.931	**0.017**
**Subjective health status**						
Excellent or very good	reference	0.292	reference	**0.001**	reference	**0.001**
Good	0.852 (0.195), 0.581–1.250	0.414	1.692 (0.224), 1.092–2.623	**0.019**	1.658 (0.222), 1.072–2.563	**0.023**
Less good or poor	0.740 (0.193), 0.507–1.079	0.117	2.510 (0.263), 1.500–4.200	**0.001**	2.644 (0.259), 1.590–4.396	**0.001**
**Employment status**						
Employed or retired but working	reference		reference			
Retired or other	0.745 (0.178), 0.526–1.056	0.098	0.772 (0.190), 0.532–1.120	0.173		
**District level SES**						
First quartile (low)	reference	**0.027**	reference	0.854		
Second quartile (moderate)	1.116 (0.200), 0.754–1.651	0.583	0.813 (0.258), 0.490–1.349	0.424		
Third quartile (high)	2.143(0.255), 1.299–3.536	**0.003**	0.909 (0.358), 0.451–1.832	0.789		
Fourth quartile (very high)	1.253 (0.219), 0.815–1.926	0.304	0.987 (0.299), 0.544–1.758	0.978		
**Gender**						
Male	reference		reference			
Female	1.319 (0.153), 0.978–1.778	0.070	1.212 (0.137), 0.863–1.702	0.267		
**Distance to intervention sites in meters**						
<800 (very low)	reference	0.150	reference	0.297		
800–1599 (low)	0.696 (0.216), 0.455–1.063	0.093	0.670 (0.246), 0.414–1.087	0.105		
1600–3499 (moderate)	0.726 (0.204), 0.486–1.083	0.117	0.858 (0.237), 0.539–1.367	0.520		
3500–5000 (high)	0.493 (0.341), 0.253–0.962	0.038	0.771 (0.389), 0.360–1.652	0.503		
>5000 (very high)	0.951 (0.249), 0.583–1.549	0.839	1.216 (0.299), 0.677–2.184	0.513		
Neighborhood setting **						
Urban	reference					
Suburban	0.676 (0.155), 0.498–0.917	**0.012**				
**Age (years)**						
60 < 65	reference	0.548				
65 < 70	0.873 (0.719), 0.213–3.572	0.850				
70 < 75	0.850 (0.715). 0.209–3.450	0.820				
75 < 80	1.131 (0.730). 0.270–4.735	0.866				
**Recruitment**						
Contacted	reference					
Volunteers	1.312 (0.167), 0.946–1.820	0.103				
**Household income**						
Low	reference	0.948				
Middle	0.939 (0.197), 0.637–1.382	0.748				
High	0.979 (0.195), 0.668–1.434	0.914				

Variable(s) entered at step number 1, Communities, Employment status, Heath status, Intervention group, Level of education, Gender, District level SES, Distance to study sites; 2, Variable removed: District level SES; 3, Variable removed: Gender; 4, Variable removed: Distance to intervention sites; 5, Variable removed: Employment status.

## Data Availability

The data presented in this study are not publicly available due to privacy issues, including the possibility of re-identification.

## Supplementary Materials

The following supporting information can be downloaded at: https://www.mdpi.com/article/10.3390/ijerph19063190/s1, Correlation Heat Map and descriptive presentations of weekly class attendance and weather expositions. Figure S1: Heat Map of correlation coefficients (Spearman’s rho). Interpretation: 0 = none, 0.1–0.2 = poor, 0.3–0.5 = fair, 0.6–0.7 = moderate, 0.8–0.9 very strong, 1 = perfect [107]. Bold type = Correlation is significant at the 0.01 level (2-tailed). NE = Neighborhood. Figure S2: Descriptive of 10 weeks’ class attendance rates. Figure S3: Weather expositions and holiday periods by days of class meeting and rate of expected attenders in different groups and communities. Note:  rainfall > 15 l/m^2^,  temperature > 30.  :wind speed > 7, IG1a: web-based intervention with subjective PA self-monitoring, first group, IG1b: web-based intervention with subjective PA self-monitoring, second group, IG2a: web-based intervention with subjective and objective PA self-monitoring, first (only) group. Table S1: Number and type of weather expositions by different groups and communities. Note: * The number of groups in each community varied according to the total number of participants in that community. The target group size was about 20 participants. ** Those who left the study before the first class were not included.
