# Supplementary material for: Ecological Predictors of Older Adults’ Participation and Retention in a Physical Activity Intervention
Source: Int J Environ Res Public Health. 2022 Mar 8;19(6):3190. doi: 10.3390/ijerph19063190 (PMC8949961; doi:10.3390/ijerph19063190)
Supplement: Supplementary file 1 [file ijerph-19-03190-s001.zip › ijerph-1625185-supplementary.pdf]

Supplementary Materials

	Community	Intervention group	District level SES	Distance to intervention sites	NE (urban/ suburban)
Community	1.000	-0.058	**-0.510**	**0.256**	**0.877**
Intervention group	-0.058	1.000	0.016	-0.072	-0.019
District level SES	**-0.510**	0.016	1.000	**-0.232**	**-0.641**
Distance to intervention sites	**0.256**	-0.072	-0.232	1.000	**0.193**
NE (urban/ suburban)	**0.877**	-0.019	**-0.641**	**0.193**	1.000

Figure S1: Heat Map of correlation coefficients (Spearman's rho). Interpretation: 0 = none, 0.1-0.2 = poor, 0.3-0.5 = fair, 0.6-0.7 = moderate, 0.8-0.9 very strong, 1 = perfect [1]. Bold type = Correlation is significant at the 0.01 level (2-tailed). NE = Neighborhood.

1. Chan, Y. Biostatistics 104: correlational analysis. *Singapore Med J* **2003**, *44*, 614-619.

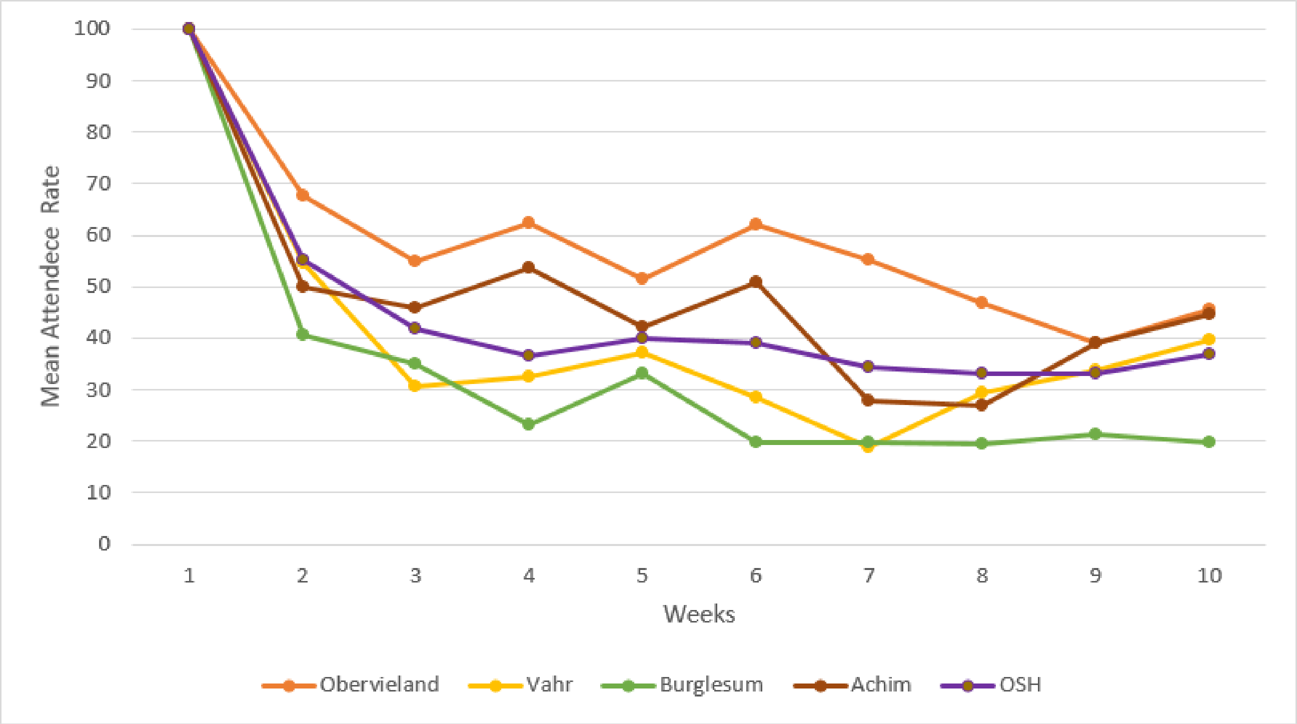

Community / Week	Week 1	Week 2	Week 3	Week 4	Week 5	Week 6	Week 7	Week 8	Week 9	Week 10
Obervieland	100	67.5	54.9	62.5	51.6	62.0	55.1	46.8	38.9	45.4
Vahr	100	54.5	30.8	32.6	37.2	28.3	18.8	29.5	33.8	39.6
Burglesum	100	40,6	35.1	23.2	33.2	19.7	19.8	19.4	21.3	19.7
Achim	100	50.0	46.0	53.5	42.2	50,9	28.0	26.8	38.9	44,8
OSH	100	55.2	41.8	36.7	39.9	39.1	34.4	33.3	33.2	36.8
**Sum**	**100**	**53.6**	**41.7**	**41.7**	**40.8**	**40.0**	**31.2**	**31.1**	**33.2**	**37.3**

Figure S2: Descriptive of 10 weeks' class attendance rates.

Table S1: Number and type of weather expositions by different groups and communities. Note: *The number of groups in each community varied according to the total number of participants in that community. The target group size was about 20 participants. ** Those who left the study before the first class were not included.

Community	Group*	Participants expected at the first class**	Date of the first class (week 2)	Date of the 10th class (week 11)	Season	N of classes with rainfall exposures	N of classes with wind speed exposures	N of classes with temperature exposures
Obervieland	1	24	08-Mar-17	10-May-17	Spring			
	2	19	22-Mar-17	24-May-17	Spring			
	3	19	29-Mar-17	31-May-17	Spring			
	4	23	05-Jul-17	06-Sep-17	Summer/Autumn		2	
	5	21	12-Jul-17	13-Sep-17	Summer/Autumn		3	
	6	21	26-Jul-17	27-Sep-17	Summer/ Autumn		2	
Vahr	1	11	23-Jun-16	25-Aug-16	Summer			1
	2	21	07-Jul-16	08-Sep-16	Summer			1
	3	24	14-Jul-16	15-Sep-16	Summer			1
Burglesum	1	15	20-May-16	21-Jul-16	Spring/ Summer	2		1
	2	14	26-May-16	28-Jul-16	Spring/ Summer	2		1
	3	23	09-Jun-16	11-Aug-16	Summer	1		1
Achim	1	23	03-Aug-17	05-Oct-17	Summer/ Autumn		3	
	2	18	17-Aug-17	19-Oct-17	Summer/ Autumn		2	
	3	23	24-Aug-17	26-Oct-17	Summer/ Autumn		2	
OSH	1	21	20-Jan-17	24-Mar-17	Winter/ Spring		1	
	2	19	27-Jan-17	31-Mar-17	Winter/ Spring		1	
	3	21	10-Feb-17	13-Apr-17	Winter/ Spring		1	
	4	19	17-Feb-17	21-Apr-17	Winter/ Spring		1	
	5	19	03-Mar-17	05-May-17	Spring		1	
**Sum**						3	19	6

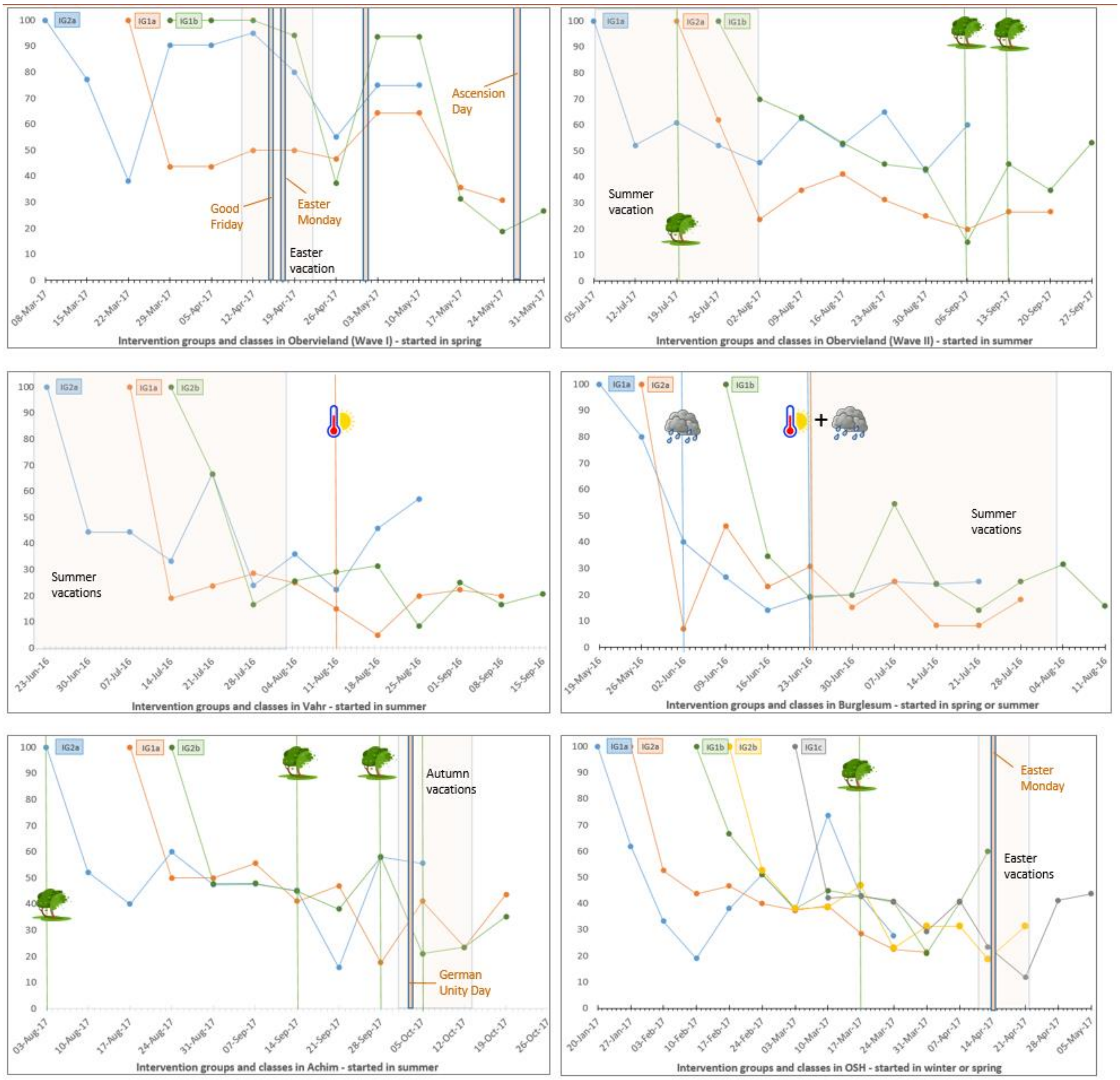

Figure S3: Weather expositions and holiday periods by days of class meeting and rate of expected attenders in different groups and communities. Note: rainfall >15 l/m2, temperature >30. : wind speed >7, IG1a: web-based intervention with subjective PA self-monitoring, first group, IG1b: web-based intervention with subjective PA self-monitoring, second group, IG2a: web-based intervention with subjective and objective PA self-monitoring, first (only) group.
